# Commentationes Societatis Regiæ Scientiarum Gottingensis

**Published:** 1781-12

**Authors:** 


### THE LONDON MEDICAL JOURNAL,

### For DECEMBER 1781.

### SECTION I.

### BOOKS.

I. *Commentationes Societatis Regiæ Scientiarum Gottingensis.* Classis Physicæ, Tomus II. ad A. 1779. 4to. Gottingæ, 1780, p. 138.

I. *Remarks on several exotic plants cultivated in the Royal Botanic Garden. By* John Andrew Murray, M. D. *Professor of Botany in the university of Gottingen.*—The plants described in this paper are as follow: 1. Rheum Hybridum, *foliis cordatis acuminatis planis, radicalibus utrinque bi-vel tridentatis, reliquis repandis.*—This plant, the seeds of which were sent from the botanic garden at Copenhagen, is supposed by our author to be a species of Mule, produced by the seminal dust of some other species of rhubarb accidentally falling on the *piſtilla* of the *Rheum Palmatum*. It is ſaid to be taller than any other rhubarb plant, and to have very large leaves, three feet long. 2. LYCIUM RUTHENICUM, *foliis faſciculatis linearibus, ramis dependentibus*.—Dr. Murray received the ſeeds of this and ſeveral other plants from Dr. Pallas, who collected them in Siberia, but he does not find any deſcription of this particular ſpecies of *Lycium* in Linnæus, Pallas, or any other botanic writer. 3. BETONICA HIRSUTA, Linn. This plant, which is indigenous in the Alps, is deſcribed by Linnæus in his 2*d Mantiſſa plant.* It has ſo much reſemblance to the *Betonica Officinalis* L. that it has been often confounded with that plant. In order to prevent a ſimilar miſtake in future, Dr. Murray gives an accurate deſcription of the *Betonica Hirſuta*, and points out the principal marks that diſtinguiſh it from the plant juſt now mentioned. 4. VERBESINA DICHOTOMA, *foliis oppoſitis ovatis tomentoſis petiolatis, caule ſupra dichotomo extimo internodio*.—Of this plant our author obſerves, that he ſhould have taken it for the *Verbeſina Lavenia* L. had not Linnæus and Burmannus both agreed in deſcribing the latter with ſmooth leaves. This and other circumſtances induce him to conſider the preſent plant as a diſtinct

ſpecies

ſpecies to which he gives the name of *Dichotoma* on account of its ſtem being divided or forked at its upper part. 5. COMMELINA BENGHALENSIS Linn. Dr. Murray is perſuaded that this plant, which is a native of Bengal, is the ſame as that deſcribed by Linnæus in his ſecond Mantiſſa under the name of *Commelina Cucullata.* He remarks at the ſame time, that in the laſt edition of the *Syſtema Vegetabilium* no mention is made of this plant, although no reaſon is given why it is omitted. 6. MALVA VIRGATA, *foliis baſi anguſtatis multiformibus partitis laciniis inciſo-crenatis pedunculis unifloris.* This plant has hitherto been conſidered as a variety of the *Malva Capenſis*, but our author is convinced that it is a diſtinct ſpecies. The engraving given of it by Dillenius in his *Hort. Eltham.* is ſaid to be inaccurate. 7. ASCLEPIAS SIBIRICA Linn.—We have here the firſt engraving that has been given of this plant, and a more minute deſcription of it than is to be found in Linnæus or any other writer.—All Dr. Murray's deſcriptions are illuſtrated by engravings.

II. *Chirurgical obſervations. By* Auguſtus Gottlieb Richter, M. D. *Profeſſor of Surgery in the univerſity of Gottingen.*—The author begins

 his his paper with lamenting the inefficacy of medicine in cafes of cancer. He is perfuaded that the remedies hitherto propofed for the cure of this difeafe have all of them done more harm than good, by wafting valuable moments ineffectually, and delaying the extirpation of the cancerous mafs till the difeafe has been beyond the reach of furgery.

Dr. Richter confiders the want of a proper attention to the diagnofis in thefe cafes as another defect in this branch of medical practice. He obferves, that modern phyficians, though extremely affiduous in difcovering new remedies aga'nft cancer, have been but little folicitous to afcertain the fymptoms and nature of the difeafe. He even does not hefitate to pronounce, that it is more difficult to determine the prefence of cancer, than it is to cure it, as, in his opinion, there is no invariable and certain criterion as yet eftablifhed, by which a cancer may be diftinguifhed from other ulcers, and he is convinced that many ulcers have been confidered as cancers, which in fact were not fuch. To this fource of error and uncertainty he afcribes the accounts given us by authors of wonderful cures performed by different remedies in cafes of cancer.

Pain, which is the moſt frequent and troubleſome ſymptom in cancerous affection, affords, in our author's opinion, no proof of the preſence of cancer, as it often occurs with great vehemence in other ſpecies of ulcers. The acrid ſanies, the callous ſtate of the lips of the wound, and the fungous excreſcence that uſually accompany cancer, are all of them rejected for the ſame reaſon.

Farther, Dr. Richter remarks that the diſeaſe happening in a glandular part is no proof of its being cancerous, as the worſt kind of cancers oftentimes occur in parts that are not glandular, while glands in different parts of the body as often ſuppurate, and heal in the kindeſt manner. He obſerves alſo, that as cancerous ulcers are not always preceded by ſchirrus, the previous exiſtence of the latter cannot be reckoned among the permanent guides to the diagnoſis in theſe caſes; and that ulcers which are not of a cancerous nature may take place in parts affected with ſchirrus, he thinks is ſufficiently proved by the firſt of the five caſes related in the paper before us.

From theſe conſiderations our author is led to conclude, that there is nothing in the origin or progreſs of the diſeaſe that can enable us to propronounce it to be of a cancerous nature. "Va-

" rious—ſays he—are the beginnings of this

" dreadful complaint. Sometimes it ariſes from

" a ſchirrus, at others from a wart, or a ſlight

" pimple. In ſome caſes it fixes its ſeat in an

" encyſted tumour; in others it begins with

" affecting ſome of the bones, and appears as a

" ſpecies of exoſtoſis or ſpina ventoſa. The ſymp-

" toms alſo of the diſeaſe vary ſo much in its

" progreſs, that the authors who have written

" on the ſubject can hardly be ſaid to have

" deſcribed one and the ſame diſeaſe. What

" definition then can we give of cancer? and

" how can we diſtinguiſh it from other ulcers

" *mali moris?*"—To theſe two queſtions the author candidly acknowledges his inability to reply.

He obſerves, that very often the term cancer, like that of malignant fever, is made uſe of as a veil to ignorance, while the cauſe of malignity lies not in the ulcer, or in the nature of the fever, but in the phyſician who treats them. He has had occaſion, he ſays, to ſee ulcers which were owing to a caries, and had been miſtakenly conſidered as cancerous; and he has ſometimes cured what was deemed an open cancer of the breaſt by repeated emetics and purges, the ill concondition of the ulcer in thofe cafes having been occafioned merely by an acrid faburra in the primæ viæ.

As a proof of the very oppofite opinions of medical authors on the fubject of cancer, Dr. Richter quotes the writings of the late Profeffor Monro of Edinburgh and Mr. Hill. The former of thefe afferts, that of fixty patients in whom he had extirpated cancers himfelf, or feen cancers extirpated by others, four only were faved, all the others dying miferably after the operation. Mr. Hill on the contrary affirms, that of eighty patients on whom he himfelf performed the fame operation, only two died.

After thefe preliminary obfervations the author gives an account of five cafes that have occurred to him in his practice. The firft is the cafe of a woman, aged 40 years, who after exceffive grief for the lofs of her hufband perceived a fmall tumour in the lower part of her left breaft, which increafed by degrees to the fize of a man's fift, and remained in that ftate for feveral years. At length, on a fudden, the whole breaft became wonderfully enlarged. It was at this period of the difeafe that our author firft faw the patient. The breaft had now acquired thrice the bulk of a man's head, and extended as low down as the navel, navel, appearing like a bladder exceſſively diſtended with water. On the right ſide a ſmall, hard lump was to be felt, near which was a ſmall ulcer that afforded a conſiderable diſcharge of a reddiſh, watery ſanies. The patient did not complain of pain when the breaſt was preſſed with a finger; nor did there appear to be any diſeaſe of the axillary glands, or any adheſion of the tumour to the pectoral muſcle. She was troubled however with hectic fever every evening, and likewiſe with profuſe ſweats, oppreſſion at the breaſt, cough, and great pain about the right ſcapula.

The author at firſt tried the effect of medicines, but theſe affording the patient no relief, he judged it neceſſary at the end of a few weeks to have recourſe to the knife. On making an inciſion into the tumour, two pints of a reddiſh fluid were diſcharged from a large cyſt formed within the ſubſtance of the breaſt. The breaſt when amputated was found to weigh eight pounds. It included two cavities, one of which was large enough to admit a man's fiſt, and was ſurrounded by a diſeaſed, pulpy maſs. This cavity had a ſmall communication with the external ulcer.

The patient died on the third day after the operaoperation, and on diffection part of the true ribs of the right fide and the middle of the fternum were found to be carious. The right cavity of the thorax was filled with a reddifh fluid, and the whole of the right lobe of the lungs difeafed, that part of it next the carious ribs being in a ftate of fuppuration. The ftomach and convex furface of the liver likewife appeared to be in an inflamed ftate.

The fubject of the fecond cafe was an unmarried woman, twenty-four years of age, who applied to our author for the cure of a fchirrous tumour in each of her breafts. That in her left breaft, which appeared firft, had come on without any blow or other external caufe, and had gradually enlarged to the fize of a man's fift, and for fix months had been attended with a conftant burning pain, though not always of equal vehemence. The fchirrus in the right breaft had begun to appear about the time the firft became painful, and was now abo1t the fize of a pigeon's egg. Both tumours were perfectly moveable; the fkin that covered them was found, and although the pain of the left breaft fometimes extended to the axilla, no induration was perceptible in the axillary glands of either fide.

 The

The general health of the patient did not appear to be affected. Both tumours were removed by the knife. On the fecond day after the operation the menfes appeared, and the day following the patient had a flight fymptomatic fever. From this period fhe had recourfe to a milk diet. On the fixth day both the wounds afforded a good pus, but of a fingular fmell. Our author compares it to that of wood in a ftate of putrefaction.

On the tenth day a tumour as large as a filberd nut appeared on the margin of the wound in the left breaft, but in a few days gradually difappeared. On the 22d the pus became thin, acrid, and fœtid, and the night following the menfes again made their appearance. On the 24th the patient was frequently attacked with fhivering, and the next day had an eryfipelatous eruption on her breaft and abdomen. The pus at the fame time was thin, like the ferum of milk, and fo extremely acrid, that it excoriated both the wounds. The patient took an ounce and a half of Glauber's falt, and in about five days the eruption difappeared and the pus was of a better confiftence; but about this time a painful tumour appeared near the lip of the wound in the left breaft, and in a few days fuppurated, purated. After this the patient recovered without any other accident. An iſſue was made in her left arm, and ſhe was adviſed to perſevere for ſome time in the uſe of a milk diet. Six months after the operation Dr. Richter was informed by letter that ſhe continued in perfect health.

The ſchirrus taken from the left breaſt was of a cartilaginous conſiſtence, but did not afford the leaſt appearance of ſuppuration.

The author having remarked that in this, as well as in many other caſes of ſchirrus or cancer of the breaſt, the diſeaſe has been preceded by grief, thinks the obſervation made by Bierchen well founded, which is, that of the cauſes prediſpoſing to cancer none are more frequent or painful than violent paſſions of the mind.

The third caſe related by our author affords an inſtance of a ſingular diſeaſe of the breaſt. The patient was a female, thirty years of age, of an irritable habit, and ſubject to profuſe menſtruation. She applied to Dr. Richter on account of a ſwelling of the ſize of a walnut in her right breaſt. From this tumour, which was perfectly moveable, there was a ſeries of ſmaller tumours extending to the nipple, each of which was about the ſize of a pea, and from the nipple to the axilla a hard chord-like appearance was to be felt, of the fize of a finger for fome way upwards, after which it gradually leffened, and at length totally difappeared in the axilla, fo that its termination could not be felt.

Whenever the larger tumour was compreffed a reddifh watery fluid iffued in confiderable quantity from the nipple, till the feries of fmaller tumours was obliterated, and the large one became flaccid and feemingly empty. In the fpace of a few hours, however, they filled again as before, and whenever they were too much diftended the patient complained of a burning pain, which was foon removed by emptying the tumour.

Previous to the firft appearance of this difeafe the patient had been fubject to a profufe hæmorrhoidal flux, which was fuddenly checked, and fome months afterwards fhe perceived that the linen covering her breaft was tinged with blood, which was found to proceed from a very fmall opening near the nipple. At length the tumour made its appearance, and gradually increafed to the fize of a hen's egg. At the end of a few months the whole breaft fwelled on a fudden to an enormous bulk, but was reftored to its former fize by the internal ufe of hemlock, leaving, leaving, however, the tumour in its original ftate. From this time the opening juft now fpoken of clofed, and the fluid began to iffue from the nipple.

Dr. Richter fuppofing this difeafe to be owing to a varicous dilatation of the veffels of the breaft, rather than to a cancerous affection, declined performing any furgical operation; but contented himfelf with prefcribing internal remedies. The patient was ftill following his prefcription at the time the paper was written, fo that we are not informed how the cafe terminated.

In the fourth cafe we have an account of a cataract attended with extreme fenfibility of the eye. The difeafe was removed by extracting the chryftalline lens.

In fpeaking of this cafe the author remarks, that formerly it was his practice not to uncover the eye till about the eighth day; but that he is now convinced by experience of the propriety and even neceffity there is of feparating and cleanfing the eye lids every day after the operation. In the cafe before us the eye lids became fwelled and painful on the third day after the operation, but this troublefome fymptom was foon removed by cleanfing the eye lids.

In

In the fifth and laſt caſe we have another in-inſtance of a cataract ſucceſsfully extracted by our author. The only remarkable circumſtance in this caſe was the diminiſhed ſize of the eye, which had loſt about a third of its original bulk previous to the operation.

III. *Anatomico-neurological obſervations on the Semilunar Ganglion and Plexus ſituated in the abdomen, and on the nerves that compoſe it. By* Henry Auguſtus Wriſberg, M. D. &c.—This elaborate paper, which does not admit of abridgement, is divided into four parts. In the firſt the author treats of the phrenic nerve; in the 2d of the 8th pair of nerves; in the 3d of the courſe of the great ſympathetic nerves through the thorax to the ſemilunar ganglions; and in the 4th of the origin, ſtructure, ſituation, figure, and ſize of the ſemilunar ganglions and plexus. The anatomical deſcriptions are illuſtrated by five engravings.

IV. *An account of ſome uncommon tumours that occur about the wriſt and in the palm of the hand, which, though ſimilar in their appearance, are very different in their nature, and of courſe require different modes of treatment. By* Olaus Acrel.—The tumours deſcribed in this paper are of two kinds. One of theſe, according to our author, orioriginates from what he terms a carcinomatous caries of ſome of the bones of the carpus, and becomes a cancerous fungus; the other, which he deſcribes as being leſs frequent and dangerous, is ſaid to be a ſpecies of atheroma, that is commonly ſituated about the joint and ligaments of the wriſt. Both theſe ſpecies, he ſays, are ſo exactly alike in their external appearance, that he has ſeldom been able to diſtinguiſh one from the other before the ſkin and tendinous faſcia with which they are covered have been removed by the knife.

That theſe tumours are rare diſeaſes he concludes from his having, in the courſe of forty years practice in every part of ſurgery, met with only three caſes of the former, and two of the latter ſpecies. Two inſtances of tumours, which ſeem to reſemble the firſt or cancerous kind, are deſcribed by Petit in his treatiſe *Des Maladies des Os*, tom. ii. p. 507; and a deſcription of two ſimilar tumours is given by Ruyſch in his *Obſerv. Anat. Chirurg.* p. 74. obſ. lxxxi. but of the ſecond or atheromatous ſpecies, a caſe related by Gooch in the ſecond volume of his caſes, p. 381, is the only inſtance that our author has been able to meet with in books.

The tumours of the firſt ſpecies, we are told, arife arise either from the junction of the bones of the fore arm with those of the carpus, or wholly from the upper row of the latter. They differ in their size, shape, and consistence, as well as in the degree of pain that attends them, but are generally situated under the tendons, and the colour of the skin remains unchanged.

The only inconveniences complained of for some time by the patient in these cases are a sense of weight in the hand, and more or less impediment to the motion of the fingers. Like other cancerous diseases they advance slowly in the beginning, but at length suddenly increase in bulk.

The three cases that have fallen under our author's observation, and which are related at large in the paper before us, serve to shew how very inadequate the resources either of physic or surgery are to the disease in question. From internal remedies no relief can be expected, and a surgical operation tends only to accelerate the death of the patient; the best way, therefore, seems to be to attempt only a palliative mode of treatment.

Two of the patients ascribed the origin of their complaint to a blow, and the third to a sprain. The subject of the first case was a married ried woman, aged 22 years. At the time ſhe applied to our author the tumour was three inches in diameter. It had begun to appear about four years before this, but during the firſt three years its growth had been extremely ſlow.

After cutting through the ſkin the whole of the diſeaſed maſs was carefully ſeparated from the ſurrounding tendons and cellular membrane, and completely extirpated without any conſiderable hæmorrhage. For ſome weeks after the operation the wound ſeemed diſpoſed to heal kindly, but at length it aſſumed an unfavourable aſpect. It became filled with a cancerous fungus, the patient was attacked with hectic fever and colliquative ſweats, and the whole hand became tumid and painful.

The amputation of the hand was now recommended as the laſt reſource, and the patient readily ſubmitted to it, but it was not long before the ſtump appeared to be in a cancerous ſtate, and ſhe died eight weeks after the operation.

The ſecond patient, whoſe caſe is related, was a man forty years of age. The tumour was ſix inches in length, four in breadth, and three in thickneſs. It was extirpated in the ſame manner as that juſt now deſcribed, and like that ter-

 minated minated in the amputation of the hand, and in the death of the patient.

The ſubject of the third caſe was a female patient in the Royal Hoſpital at Stockholm. The tumour was flattened at the top and of conſiderable bulk. It adhered to the carpus by a kind of peduncle, and ſeemed to be free from any adheſion to the tendons, which, inſtead of paſſing over it as in the former caſes, were to be felt on each ſide of it. After dividing the integuments a ligature was paſſed round the baſis of the tumour, which dropped off in about ſeven weeks. At the end of four months the patient left the hoſpital with her hand in a cancerous ſtate. We are not informed of the event of the caſe.

Of the atheromatous tumour only a ſingle inſtance is related. The author ſuppoſes this ſpecies to be originally formed by a diſtenſion of ſome part of the capſular ligament of the bones of the carpus inſinuating itſelf by degrees under the muſcles, tendons, and aponeuroſes of the palm of the hand, and forming a ſeries of minute cyſts communicating with one another and filled with concreted mucus. In the caſe here deſcribed the diſeaſe was ſuppoſed to have been brought on by a contuſion, and the tumour by by its bulk totally impeded the motion of the fingers. Upon making an incision into it only a small portion of inspissated mucus was discharged, and it was therefore deemed expedient to pass five setons through different parts of the tumour. These were kept in during fourteen weeks, but at the end of that time, although the suppuration had been constant and considerable, the disease was far from being removed, as a great part of the cyst was still remaining, and seemingly in a callous state, so as to be incapable of further suppuration. At this period of the treatment our author ventured to inject into the fistulous sinuses formed by the setons an irritating liquor, prepared by diluting the marine acid with water. This produced considerable inflammation of the hand and arm followed by a copious suppuration and large sloughs, so that in about six weeks more the whole of the cyst was thought to be removed, and the patient had recovered the use of her hand and fingers.

